# Evaluation of Flat Sheet UF PES Membranes Modified with a Polymerized Coating of Bicontinuous Microemulsion for Wastewater Treatment: Insights from Laboratory MBR Experiments

**DOI:** 10.3390/membranes16010024

**Published:** 2026-01-02

**Authors:** Sneha De, Tran Ly Quynh, Francesco Galiano, Raffaella Mancuso, Bartolo Gabriele, Jan Hoinkis, Alberto Figoli

**Affiliations:** 1 Center of Applied Research (CAR), Karlsruhe University of Applied Sciences (HKA), Moltkestrasse 30, 76133 Karlsruhe, Germany; v.quynhtl2@vinfast.vn; 2 Institute on Membrane Technology (ITM-CNR), Via P. Bucci 17c, 87036 Rende, CS, Italy; f.galiano@itm.cnr.it (F.G.); a.figoli@itm.cnr.it (A.F.); 3 Laboratory of Industrial and Synthetic Organic Chemistry (LISOC), Department of Chemistry and Chemical Technology (CTC), University of Calabria, Via P. Bucci 12/C, 87036 Rende, CS, Italy; raffaella.mancuso@unical.it (R.M.); bartolo.gabriele@unical.it (B.G.)

**Keywords:** ultrafiltration (UF), polymerizable bicontinuous microemulsion (PBM), membrane bioreactor (MBR), wastewater treatment (WT), model wastewater (MW)

## Abstract

The study investigates the performance of polyethersulfone (PES) ultrafiltration (UF) membranes modified with a coating of polymerizable bicontinuous microemulsion (PBM) for membrane bioreactor (MBR) applications. Two types of PBM-modified PES membranes—casting-coated and spray-coated—were compared with a commercial PES membrane. A laboratory side-stream MBR (ssMBR) was employed to treat model wastewater (MW) with activated sludge under aerobic conditions. The fouling propensity of the membranes in ssMBR was evaluated through the implementation of two protocols: (i) flux-step test to treat low-strength domestic model wastewater (DMW) and (ii) constant flux test to treat high-strength olive mill model wastewater (OMW). The findings indicated that both the commercial PES and PBM spray-coated PES membranes started to critically foul at 36 L m^−2^ h^−1^. The PBM spray-coated membranes showed enhanced fouling resistance in comparison to the PBM casting-coated membranes. The deposition of the biofouling layer was the thinnest on PBM spray-coated membranes, which can be attributed to the low surface charge and high hydrophilicity of the modified membrane surface. In contrast, deposition of a thicker fouling layer was found on the commercial PES membrane, which can be attributed to the relatively higher surface charge promoting organic adsorption. A comparison of the fouling trends exhibited by commercial PES and PBM spray-coated membranes in OMW treatment revealed that they have similar fouling tendencies. However, a notable distinction emerged when the PBM spray-coated membrane was observed to demonstrate a lower fouling propensity accompanied by comparatively thinner fouling layers. The results demonstrate that the PBM spray-coated membranes have enhanced fouling resistance and filtration efficacy in MBRs treating wastewater with diverse strengths, thereby affirming their potential for application in wastewater treatment systems.

## 1. Introduction

There is a growing interest today in advanced wastewater treatment (WT) technologies capable of producing high-quality effluents suitable for reuse [1,2]. In this regard, membrane bioreactors (MBRs) have gained attention due to their compact design, superior effluent quality, and ability to handle high mixed liquor suspended solids (MLSS) concentrations [3,4]. However, membrane fouling remains one of the most significant operational challenges for MBR technology in WT [5,6]. Membrane fouling leads to reduced membrane performance, increased energy demand, and consequently higher operational costs. Consequently, significant research has been conducted on antifouling and high-performance ultrafiltration (UF) membranes, focusing on materials and engineering surfaces. These modifications involve: (i) chemical surface modification [7], (ii) polymer blending [8], (iii) incorporation of nanomaterials such as TiO_2_ and graphene oxide [9,10], (iv) hydrophilization [11,12], (v) zwitterionic coatings [13,14], and (vi) engineered surface structures such as surface patterning [15].

Another promising technique is the modification using polymerizable bicontinuous microemulsion (PBM) materials. PBMs have recently emerged as a promising platform for engineering hydrophilic, antifouling, and functionally tailored membrane surface layers [16,17,18,19,20,21]. PBM-based coatings have been shown to enhance surface wettability, suppress organic adsorption, and improve chemical resistance—properties that are critical for achieving stable and sustainable wastewater treatment performance. Spray-coating methodologies enable the uniform application of microemulsion layers onto flat-sheet PES substrates, offering precise control over coating thickness while ensuring robust adhesion following polymerization [21,22]. The resulting PBM layer functions as an effective protective barrier that promotes flux recovery and mitigates membrane fouling. When applied to UF-grade PES coatings, it significantly increases hydrophilicity and produces smoother surface morphologies, thereby reducing foulant deposition and facilitating cleaning [21]. Moreover, PBM formulation can incorporate functional groups—such as hydroxyl or carboxyl moieties, that modulate electrostatic interactions with foulants, providing an additional mechanism for antifouling enhancement. Accordingly, elucidating the relationships among coating composition, microstructural characteristics, and MBR operational performance is essential for guiding the rational design of next-generation membranes.

A study by De et al. (2023) [21] assessed the influence of the PBM-coating method and demonstrated the potential of PBM spray-coated membranes over casting-coated and commercial UF PES membranes for use in MBRs. The critical flux was found to be approximately 40% higher for the PBM spray-coated membrane than the commercial PES membrane, using humic acid as a model foulant [21]. This study aims to build on these findings by investigating the performance of PBM spray-coated PES membrane under realistic conditions in a laboratory side-stream MBR (ssMBR) using model wastewaters (MW) such as low-strength domestic wastewater (DMW) and high-strength olive mill wastewater (OMW). Domestic wastewater is the most important type of wastewater worldwide and therefore offers great market potential for application. OMW is considered one of the most challenging industrial effluents for membrane filtration due to its high-fouling potential resulting from a combination of chemical, physical, and biological characteristics [23].

## 2. Materials and Methods

### 2.1. Membrane Preparation

The commercial Flat sheet Nadir^®^ UP150 membrane (Mann+Hummel International GmbH, Ludwigsburg, Germany) was the first membrane sample tested in this study [24,25]. Its membrane surface was modified by application of a PBM layer to obtain 2 different samples—PBM casting-coated membranes and PBM spray-coated membranes [21]. A 4 µm thick casting layer of PBM was manually applied on the PES membrane surface using a spiral casting knife, TQC sheen AB3050 (Industrial Physics, Essen, Germany) to obtain the PBM casting-coated membrane [26]. The PBM was sprayed onto the PES membrane surface using a homemade spray-coating system to obtain the PBM spray-coated membrane [21]. The detailed preparation of the 3 different membrane samples for testing has been described in our previous work [21]. The PES membranes were modified in inert conditions. They were ready to be used after photopolymerization of the coated bicontinuous microemulsion by ultraviolet (UV) light (300 mW cm^−2^) within 1 min. The membrane sheets were cut with an active filtration area of 84 cm^2^ to fit the crossflow module of the laboratory side-stream MBR (ssMBR). The membrane cut-outs were wetted in deionized (DI) water for 3 h before installation in the ssMBR.

### 2.2. Laboratory Side-Stream MBR (ssMBR)

**Figure 1:**
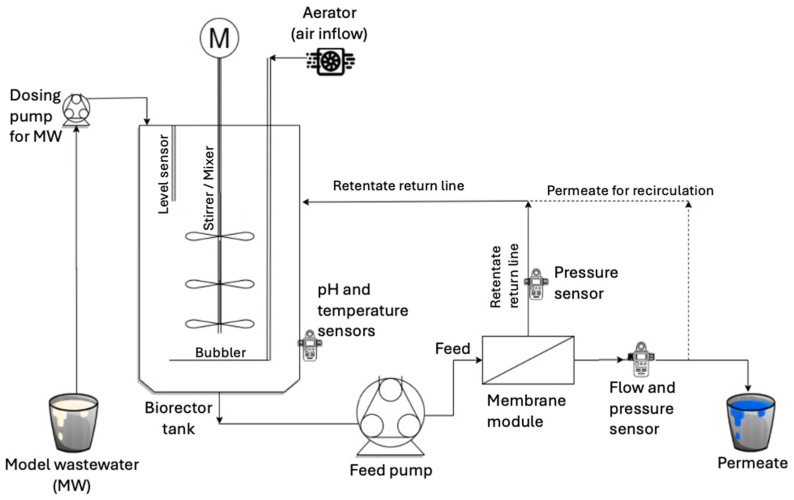
Schematic illustration of laboratory ssMBR used to test the membrane performance.

A stainless steel fermentor, BioStat^®^ C-DCU from Satorius AG (Goettingen, Germany), was used as the ssMBR to perform experiments with MW [27,28]. All the data was monitored by the in-built sensors of the BioStat C-DCU system and collected by the supervisory control and data acquisition system. The 30 L bioreactor tank was filled with 20 L of activated sludge (Municipal Sewage Treatment Plant, Germersheim, Germany) to treat the MW. The prepared MW was slowly added to the bioreactor tank at a rate of 100 mL h^−1^, ensuring that the foam was contained within the tank (Figure 1). The MW was mixed with the sludge by a stirrer continuously rotating at 200 rpm. This prevented the settling of any excess sludge at the bottom of the bioreactor tank.

The MBR was acclimated for 10 days, considering the hydraulic retention time (HRT) of 8.5 days. The HRT was long due to the low throughput associated with the small membrane size used in the membrane module. The MBR was operated with a recirculation flow rate of 28 L h^−1^, pH of 6.5–7.5, and within a temperature range of 20–24 °C. The MBR produced 2.0–2.5 L day^−1^ of permeate during its acclimation phase. Mixed liquor suspended solids (MLSS) concentration in the range of 4.0–6.0 g L^−1^ was maintained in the bioreactor tank during the experiments. The activated sludge was released after the experiments with one type of MW were completed. The MBR was cleaned with a 10% hydrogen peroxide (H_2_O_2_) solution, followed by thorough rinsing with water to remove any trace of chemicals or cleaning agent, before loading a fresh batch of activated sludge for treatment of MW.

### 2.3. Model Wastewater (MW) Preparation

A fresh batch of MW was prepared for the experiments, ensuring the MW characteristics were consistent. The DMW was prepared to replicate low-strength domestic wastewater, which is similar to diluted urban greywater (Table 1, Table 2, Table 3 and Table 4) [29,30]. The OMW was prepared to mildly replicate the characteristics of typical high-strength olive mill wastewater (Table 2, Table 3, Table 4 and Table 5). It comprised commonly found minerals in olive mill wastewater, such as potassium, ammonium, and chloride, in addition to 2 phenolic compounds—tannic acid (relatively higher molecular weight) and vanillic acid (relatively lower molecular weight) to increase the organic load of the MW (Table 3) [31,32,33,34]. The pH of the prepared DMW was 7.0 ± 0.5, whereas the pH of the prepared OMW was 5.2 ± 0.5. The wastewater samples were characterized using spectrophotometric cell tests (Spectroquant^®^ NOVA 60A) for chemical oxygen demand (COD), ammonium (N-NH_4_^+^), nitrate (N-NO_3_^–^), and orthophosphate (P-PO_4_^3–^) (Table 3). TOC-L CPH/CPN (Shimadzu, Darmstadt, Germany) was used to measure the total organic carbon (TOC).

### 2.4. Characterization Methods for Membranes

The fouled membranes were lightly rinsed in DI water, ensuring that the large particulates from the sludge that had deposited on the surface of the fouled membranes were released. The fouling layer was retained on the membrane surface undisturbed. The membranes were then dried at room temperature before analyzing. The details of the characterization tools used to analyze the fouled membranes are described in our previous work (Table 6) [21].

### 2.5. Fouling Test in ssMBR

The membrane samples were subjected to 2 fouling test procedures in the laboratory ssMBR: (i) a flux-step test using low-strength DMW, and (ii) a constant flux test using high-strength OMW.

The flux-step protocol, adopted from the work of Clech and Jefferson et al. [35], produced data for comparing the fouling behavior of membranes by observing the changes in TMP at different flux levels. The ssMBR was initially operated at a constant flux of 12 L m^−2^ h^−1^ for 60 min to obtain a stable throughput. The flux was then increased in increments of 12 L m^−2^ h^−1^ at 25-min time intervals (see Section 3.2.1). Only ascending flux-step increments were carried out because critical fouling of the membranes resulted in: (i) an unstable recirculation flow in the ssMBR, and (ii) a steep increase in TMP (the rate of increase in TMP with respect to time, dP/dt ≥ 4 mbar min^−1^). The steep increase in the TMP indicated the start of critical membrane fouling and the onset of permeability loss, implying that the membrane should be cleaned [35]. The operating TMP safety threshold of the ssMBR was set to 2 bar to prevent any membrane damage during the test.

The constant flux test was conducted at 12 L m^−2^ h^−1^, and the increase in TMP of the membranes was observed until it reached the safety threshold of 2 bar (see Section 3.2.2).

## 3. Results and Discussion

### 3.1. Membrane Morphology and Properties

The characterization and pure water permeability (PWP) of the membranes are published in our previous work [21].

### 3.2. Fouling Experiments

#### 3.2.1. Flux-Step Test with Low-Strength DMW

**Figure 2:**
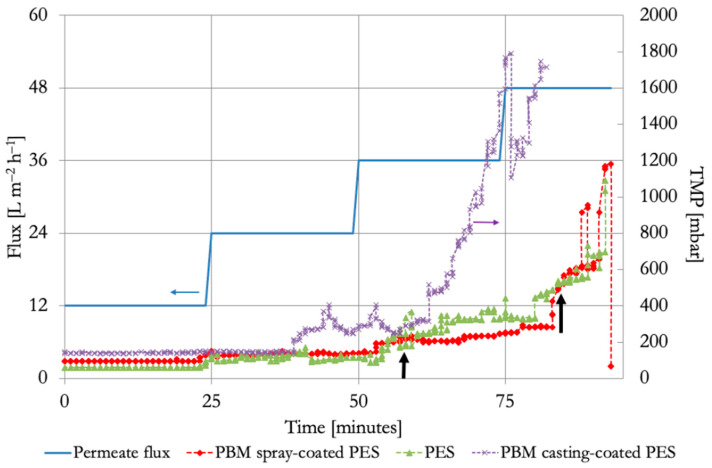
Flux versus TMP curve from flux-step test in laboratory ssMBR treating low-strength DMW using commercial PES membrane, PBM casting-coated PES membrane, and PBM spray-coated PES membrane. Onset of critical fouling of commercial PES membrane (left black arrow) and PBM spray-coated membrane (right black arrow).

The commercial PES membrane has the lowest TMP (~80 mbar) of the 3 membranes at an initial flux of 12 L m^−2^ h^−1^ (Figure 2). Subsequently, the TMP of the PBM spray-coated membrane is marginally elevated (~90 mbar), followed by the PBM casting-coated membrane, with the highest initial TMP (~130 mbar). The modification of the PES membranes with an additional PBM-coated layer increased the inherent membrane resistance, which led to relatively higher TMP and reduced permeability [21].

The initial permeability of the commercial PES membrane at a flux of 12 L m^−2^ h^−1^ was 152 L m^−2^ h^−1^ bar^−1^. The permeability of the PBM casting-coated membrane and PBM spray-coated membrane at a flux of 12 L m^−2^ h^−1^ was 90 L m^−2^ h^−1^ bar^−1^ and 130 L m^−2^ h^−1^ bar^−1^, respectively. This shows that the PBM coating causes loss in permeability even at low flux operation in MBR, while adding low-fouling benefits to the membrane.

**Figure 3:**
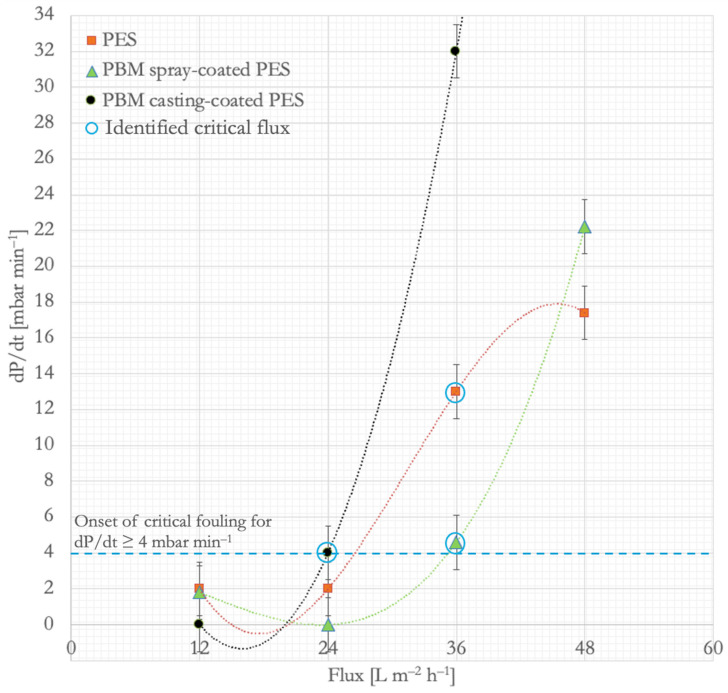
Plot of dP/dt versus flux to identify the onset of critical fouling in commercial PES membrane, PBM casting-coated PES membrane, and PBM spray-coated PES membrane in a laboratory ssMBR treating low-strength DMW.

At the flux-step of 24 L m^−2^ h^−1^, the commercial PES and PBM spray-coated PES membranes reached the same TMP of about 150 mbar, whereas the TMP of the PBM casting-coated membrane increased above 200 mbar. This shows that commercial PES and PBM spray-coated membranes are better suited for MBR applications than the PBM casting-coated membranes because the typical design flux range of a submerged MBR is 15–25 L m^−2^ h^−1^ [3,5]. At a flux step of 36 L m^−2^ h^−1^, the rate of increase in TMP (dP/dt) for all the membranes exceeded the threshold of 4 mbar min^−1^, indicating an inflection point on the dP/dt versus flux plot, when the membrane fouling escalates (Figure 3). The onset of critical fouling of the commercial PES, PBM casting-coated, and PBM spray-coated membranes was identified at 36 L m^−2^ h^−1^, 24 L m^−2^ h^−1^, and 36 L m^−2^ h^−1^, respectively (dP/dt ≥ 4 mbar min^−1^). This trend is similar to the critical fluxes of the membranes determined in our previous study [21]. This showed a 50% improvement in the performance of the PBM spray-coated membrane compared to the PBM casting-coated membrane with regard to critical flux. Despite having the same critical flux, the low-fouling behavior of the PBM spray-coated membrane is demonstrated by a lower dP/dt value than the commercial PES membrane.

#### 3.2.2. Constant Flux Test with High-Strength OMW

**Figure 4:**
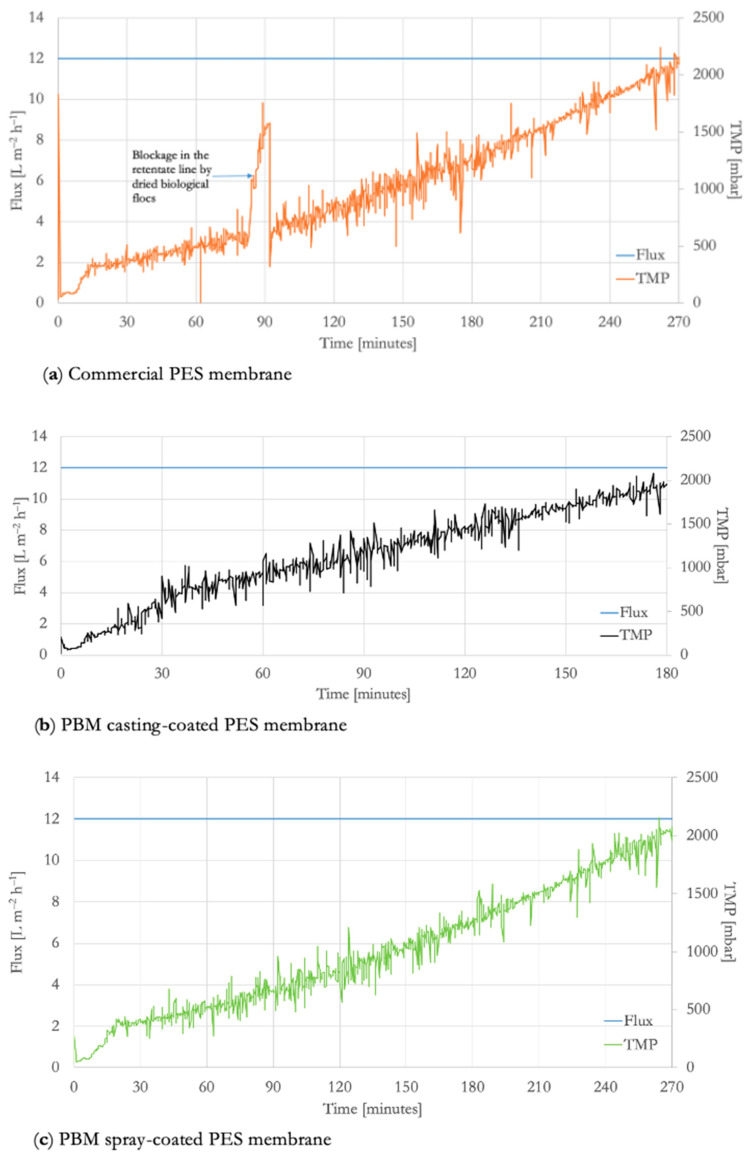
Constant flux versus TMP curve of (**a**) commercial PES membrane, (**b**) PBM casting-coated PES membrane, and (**c**) PBM spray-coated PES membrane in a laboratory ssMBR treating high-strength OMW.

The severity of membrane fouling resulting from the treatment of high-strength OMW in ssMBR was evident from the linear increase in TMP with the passage of time while operating at a constant low flux of 12 L m^−2^ h^−1^ (Figure 4). The membranes fouled steadily such that the TMP reached 2 bar (safety threshold set point of the laboratory ssMBR) in less than 5 h during the constant flux test. Thus, a flux-step protocol could not be implemented in the ssMBR treating high-strength MW.

The TMP of the PBM casting-coated membrane increased to 2 bar within 2 h of operation, whereas the commercial PES and PBM spray-coated membranes operated for 4.5 h. The increase in TMP over a relatively shorter time duration is an indicator of membrane fouling [36], which implies that the PBM casting-coated membrane is not suitable for MBRs treating high-strength wastewater. The dP/dt of the PBM casting-coated membrane was 12 mbar min^−1^, whereas the dP/dt of the commercial PES and PBM spray-coated membrane was 7.5 mbar min^−1^. This corroborates the membrane fouling trends observed in experiments with humic acid solution in our previous study [21] and with low-strength DMW in the ssMBR (See Section 3.2.1), where the fouling progression of PBM spray-coated and commercial PES membranes is similar. This test confirms that the thickness of the added low-fouling layer represents a tradeoff in achieving effective performance in real scenarios of wastewater treatment using MBR.

### 3.3. Characterization of Fouled Membranes

**Figure 5:**
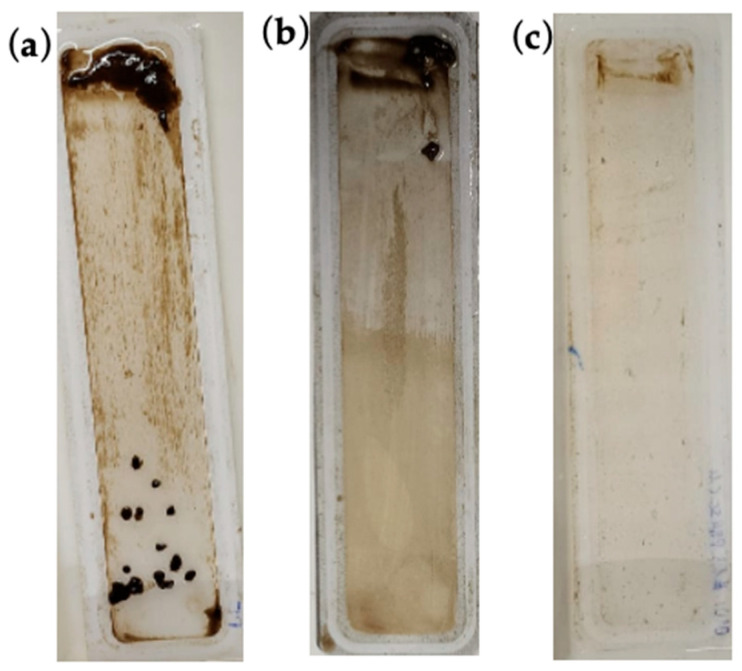
Visual impression of (**a**) commercial PES membrane, (**b**) PBM casting-coated PES membrane, and (**c**) PBM spray-coated PES membrane fouled by treatment of low-strength DMW in laboratory ssMBR.

**Figure 6:**
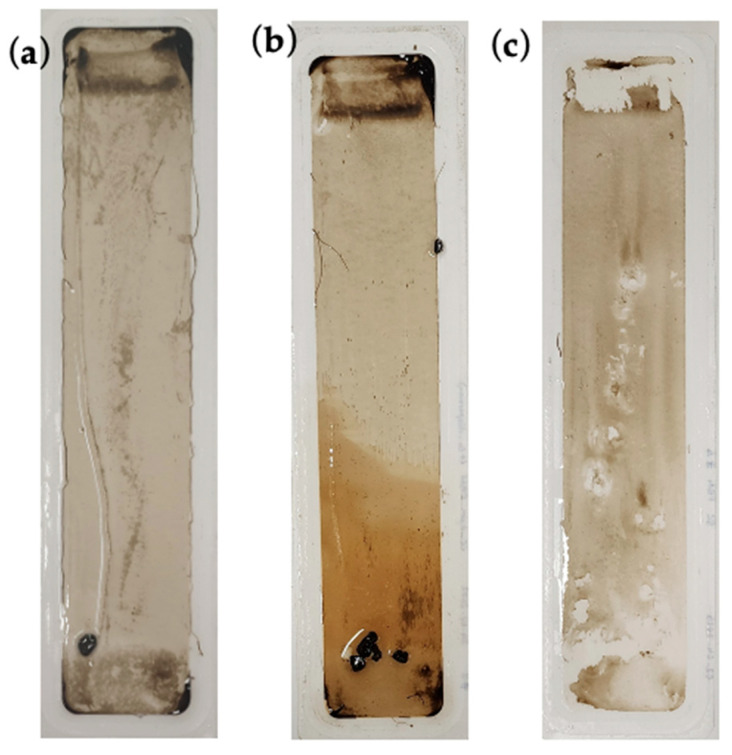
Visual impression of (**a**) commercial PES membrane, (**b**) PBM casting-coated PES membrane, and (**c**) PBM spray-coated PES membrane fouled by treatment of high-strength OMW in laboratory ssMBR.

The fouling layer on the membranes was visibly indicated by the biofouling layer deposited on the membrane surface (Figure 5 and Figure 6). It can be inferred from the visual impression of the fouled membranes that the PBM spray-coated membrane had fewer impurities on its surface than the commercial PES and PBM casting-coated membranes.

The larger particulates on the membrane are the biological flocs that comprise the activated sludge used in the bioreactor for degradation of the wastewater (feed). These biological flocs are composed of EPS, SMP, bacteria, etc., which are part of the MLSS (sludge characteristics) of the MBR [37]. The tendency of these biological flocs to adsorb on the PES and PBM casting-coated surface appeared greater than on the PBM spray-coated membrane, based on the visual appearance of the fouled membranes. The ochre yellowish shade on a portion of the PBM casting-coated membrane in Figure 6b is indicative of the fouling layer of OMW. The OMW inherently possessed a dark brown color due to the presence of tannic acid in its composition.

#### 3.3.1. Detecting PBM on Fouled Membranes

**Figure 7:**
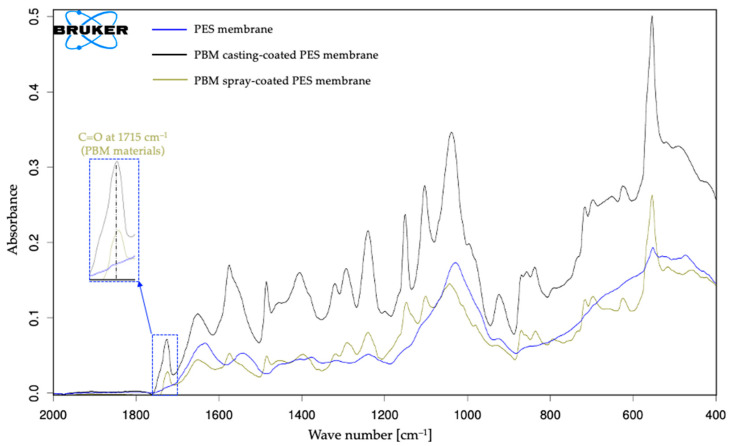
ATR-FTIR spectra of commercial PES membrane, PBM casting-coated PES membrane, and PBM spray-coated PES membrane fouled by treatment of low-strength DMW in laboratory ssMBR.

The deposition of a fouling layer on the membranes was indicated by the attenuated absorbance amplitude in the ATR-FTIR spectral curve (Figure 7). The peaks of the fouled membranes were dampened by the presence of a fouling layer on the membrane surface. It is mostly clearly seen in the spectrum of the fouled PES membrane. Most of the absorbance peaks for the PBM-coated membranes were detected, but the overall spectrum of the fouled membranes differed from that of the unfouled membranes due to the presence of a fouling layer.

The presence of PBM materials on the fouled membranes was confirmed by the IR absorbance peak at 1715 cm^−1^. These peaks are detectable within the wavenumber range of 1715–1726 cm^−1^. This slight shift in the peaks could be due to the C=O stretching caused by the attachment of the fouled compounds on the membrane surface.

#### 3.3.2. CAM of Fouled Membranes

The degradation of MW by activated sludge in MBR produces a more hydrophobic biofouling layer comprising protein-rich components, lipids, amino acid residues, etc., [38,39]. The CA of the PES membrane fouled by DMW (83°) and OMW (77°) increased from its value in the unfouled or pristine state (73°), which shows that the fouled layer on the PES membrane was hydrophobic in nature. This indicates that the uncoated PES membrane is more susceptible to fouling caused by the hydrophobic fraction of wastewater [40].

On the contrary, the CA values of the fouled PBM-coated membranes were lower than their unfouled counterparts (Table 7 and Table 8). This highlights the low-fouling property of the PBM-coated membranes and indicates that the relatively hydrophilic PBM-coated surface is prone to reduced interaction with hydrophobic moieties.

The CA of the PBM-coated membranes fouled with OMW is higher than that with DMW, indicating that the biofouling caused by OMW is more severe than that caused by DMW. It was further confirmed by microscope images of the biofouling layer (Section 3.3.3).

#### 3.3.3. SEM Images of Fouled Membranes

**Figure 8:**
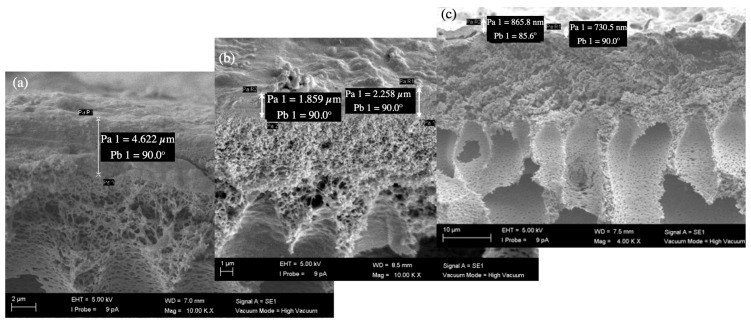
SEM micrograph of the cross-section of (**a**) a commercial PES membrane (magnification 10,000x), (**b**) a PBM casting-coated PES membrane (magnification 10,000x), and (**c**) a PBM spray-coated PES membrane (magnification 4000x) fouled by low-strength DMW.

**Figure 9:**
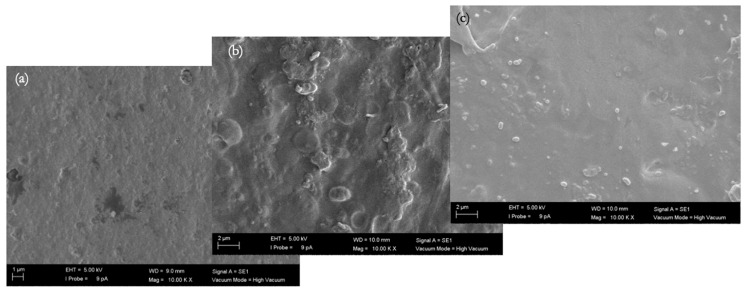
SEM micrograph (magnification 10,000x) of the top-view of (**a**) a commercial PES membrane, (**b**) a PBM casting-coated PES membrane, and (**c**) a PBM spray-coated PES membrane fouled by low-strength DMW.

**Figure 10:**
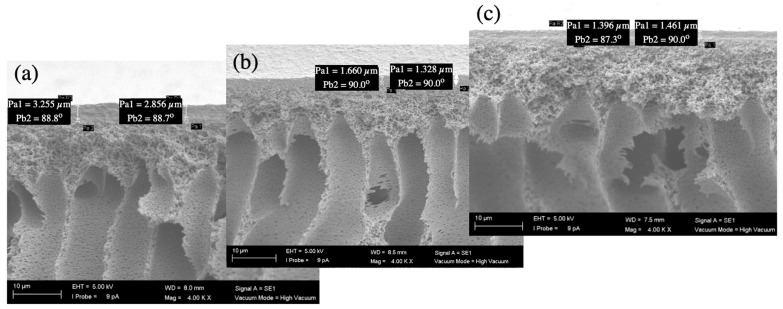
SEM micrograph (magnification 4000x) of the cross-section of (**a**) a commercial PES membrane, (**b**) a PBM casting-coated PES membrane, and (**c**) a PBM spray-coated PES membrane fouled by high-strength OMW.

**Figure 11:**
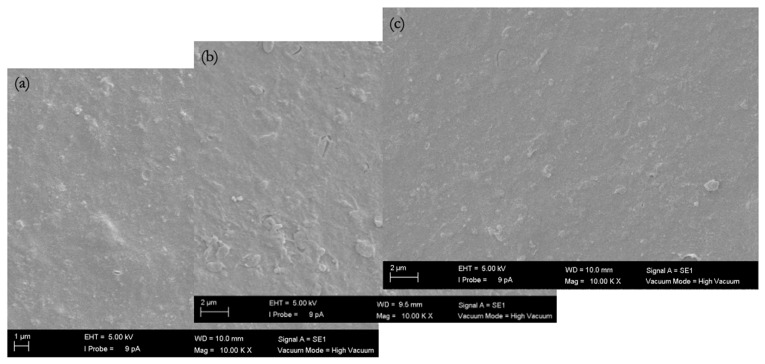
SEM micrograph (magnification 10,000x) of the top-view of (**a**) a commercial PES membrane, (**b**) a PBM casting-coated PES membrane, and (**c**) a PBM spray-coated PES membrane fouled by high-strength OMW.

The biofouling layer dried on the membrane surface was visible under an SEM in the cross-sectional views (Figure 8, Figure 9, Figure 10 and Figure 11). The thickness of the biofouling layer by DMW (4.0 ± 0.8 µm) and OMW (3.0 ± 0.6 µm) was thickest on the commercial PES membrane. This can be attributed to the relatively high surface charge, which results in the deposition of organics [17].

A thinner fouling layer was found to be deposited in the PBM casting-coated membrane fouled with DMW (2.1 ± 0.6 µm) and OMW (1.4 ± 0.2 µm). This showed the anti-fouling behavior of the PBM layer, attributed to its high hydrophilicity and low surface charge [17]. The PBM spray-coated membranes were least fouled, as evident from the thin fouling layer caused by DMW (0.7 ± 0.2 µm) and OMW (1.2 ± 0.3 µm) (Table 9). The efficacy of the spray-coated PBM layer is noticeable through the reduced thickness of biofouling in these SEM micrographs.

#### 3.3.4. Permeate Quality

The PBM-coated membranes and commercial PES membranes produced satisfactory permeate quality (Table 10 and Table 11). The pH of the permeate produced was found to be consistent with the pH of the sludge in the MBR tank.

The rejection of the membranes is improved moderately by the application of spray-coated PBM. A thicker PBM layer offers higher rejection of the organics. Thus, improving the permeate quality of the wastewater treatment process.

## 4. Conclusions

This study advances our earlier work on PBM-coated PES membranes [21] by validating their performance under MBR operating conditions. The results confirm the advantages of the spray-coating approach, particularly in achieving a thin and effective PBM layer that enhances membrane performance without substantially compromising the permeability. The key findings of this study are as follows:

- Stable performance under variable wastewater strengths.
- Enhanced anti-fouling characteristics.
- Improved permeability at lower TMP.
- Comparable baseline performance with added benefits.
- Effectiveness of the spray-coating modification strategy.

PBM spray-coated membranes maintained prolonged operational stability during the treatment of both low-strength and high-strength MW, demonstrating suitability for diverse MBR applications. The PBM spray-coated membranes exhibited a noticeably thinner biofouling layer compared with PBM casting-coated and commercial PES membranes, indicating reduced fouling propensity. The reduced PBM coating thickness facilitated higher membrane permeability, contributing to lower operating TMP and suggesting the potential for decreased energy consumption during permeate extraction. While the commercial PES membrane performed similarly in terms of critical flux and overall filtration behavior, the PBM spray-coated membrane offered incremental improvements in fouling resistance. The results reinforce spray-coating as a practical and efficient approach for applying PBM coatings to UF membranes, providing a favorable balance between fouling mitigation and hydraulic performance. It offers a promising and operationally advantageous option for MBR-based WT. Future work will focus on evaluating the modified membranes in full-scale treatment facilities and on exploring further functional enhancements, including antibacterial and antiviral properties.

## 5. Patents

The composition of PBM used for membrane coating in this work is protected by a European patent granted in 2019 [41].

## Figures and Tables

**Table 1:** Chemicals used for low-strength DMW preparation.

Chemicals	Molecular Formula	Concentration (mg L^−1^)	CAS Number, Supplier
Glucose	C_6_H_12_O_6_	640	50-99-7, Sigma Aldrich (Merck KGaA, Darmstadt, Germany)
Calcium chloride dihydrate	CaCl_2_·2H_2_O	195	10035-04-8, Supelco (Merck KGaA, Darmstadt, Germany)
Sodium bicarbonate	NaHCO_3_	220	144-5-8, Supelco (Merck KGaA, Darmstadt, Germany)
Urea	(NH_2_)_2_CO	80	57-13-6, Sigma Aldrich (Merck KGaA, Darmstadt, Germany)
Ammonium chloride	NH_4_Cl	98	12125-02-9, Carl Roth (Karlsruhe, Germany)
Trisodium phosphate dodecahydrate	Na_3_PO_4_·12H_2_O	96	10101-89-0, Supelco (Merck KGaA, Darmstadt, Germany)

**Table 2:** Chemicals used for high-strength OMW preparation.

Chemicals	Molecular Formula	Concentration (mg L^−1^)	CAS Number, Supplier
Vanillic acid	C_8_H_8_O_4_	250	121-34-6, Sigma Aldrich (Merck KGaA, Darmstadt, Germany)
Tannic acid	C_76_H_52_O_46_	250	1401-55-4, Sigma Aldrich (Merck KGaA, Darmstadt, Germany)
Glucose	C_6_H_12_O_6_	2250	50-99-7, Sigma Aldrich (Merck KGaA, Darmstadt, Germany)
Sodium hydroxide	NaOH	50	1310-73-2, Sigma Aldrich (Merck KGaA, Darmstadt, Germany)
Ammonium chloride	NH_4_Cl	100	12125-02-9, Carl Roth (Karlsruhe, Germany)
Potassium chloride	KCl	200	7447-40-7, Supelco (Merck KGaA, Darmstadt, Germany)

**Table 3:** Cell tests used for water analysis.

Parameter	Cell Test Kit	Measurement Range (mg L^−1^)	Standard Deviation (mg L^−1^)
COD	114541 (Merck KGaA, Darmstadt, Germany)	25–1500	±4.700
N–NH_4_^+^	114544 (Merck KGaA, Darmstadt, Germany)	0.5–16.0	±0.400
N–NO_3_^–^	114542 (Merck KGaA, Darmstadt, Germany)	0.5–18.0	±0.013
P–PO_4_^3–^	100474 (Merck KGaA, Darmstadt, Germany)	0.05–5.00	±0.024

**Table 4:** Chemical composition of low-strength DWM used in the experiments.

Parameters	Concentration (mg L^−1^)
COD	694.0 ± 3%
TOC	225.0 ± 2%
N–NH_4_^+^	26.5 ± 4%
P–PO_4_^3–^	8.1 ± 5%

**Table 5:** Chemical composition of high-strength OWM used in the experiments.

Parameters	Concentration (mg L^−1^)
COD	3310.0 ± 7%
TOC	1280.0 ± 8%
N–NH_4_^+^	49.5 ± 5%

**Table 6:** List of tools used for membrane characterization.

Characterization Method	Equipment and Manufacturer
Attenuated Total Reflectance Fourier Transform Infrared (ATR-FTIR) Spectroscopy	Bruker Tensor II (Ettlingen, Germany)
Contact Angle Measurement (CAM)	OCA 15EC setup, Data Physics Instruments (Filderstadt, Germany)
Scanning Electron Microscopy (SEM)	Zeiss EVO, MA100 (Assing S.p.A., Monterotondo, Italy)

**Table 7:** CAM of membranes fouled with low-strength DMW in ssMBR.

Membrane Surface	CAM (Average)	Change in CA After Fouling
PES	83° ± 4°	Increased
PES casting-coated with PBM	28° ± 3°	Decreased
PES spray-coated with PBM	26° ± 3°	Decreased

**Table 8:** CAM of membranes fouled with high-strength OMW in ssMBR.

Membrane Surface	CAM (Average)	Change in CA After Fouling
PES	77° ± 4°	Increased
PES casting-coated with PBM	50° ± 3°	Decreased
PES spray-coated with PBM	54° ± 2°	Decreased

**Table 9:** Thickness of the fouling layer obtained after fouling tests in ssMBR.

Fouled Membrane Surface	Post Flux-Step Test with Low-Strength DMW	Post Constant Flux Test with High-Strength OMW
PES	4.0 ± 0.8 µm	3.0 ± 0.6 µm
PES casting-coated with PBM	2.1 ± 0.6 µm	1.4 ± 0.2 µm
PES spray-coated with PBM	0.7 ± 0.2 µm	1.2 ± 0.3 µm

**Table 10:** Permeate quality obtained after treatment of low-strength DWM.

Parameters	Concentration in Permeates from Different Membrane Samples (mg L^−1^)		
	PES	PBM Casting-Coated	PBM Spray-Coated
COD	94.0 ± 3%	74.6 ± 4%	83.5 ± 5%
TOC	34.0 ± 2%	12.4 ± 3%	15.2 ± 4%
N–NH_4_^+^	8.5 ± 4%	8.1 ± 3%	8.4 ± 3%
N–NO_3_^–^	99.0 ± 6%	99.4 ± 4%	99.3 ± 4%
P–PO_4_^3–^	5.3 ± 4%	5.0 ± 2%	5.2 ± 2%

**Table 11:** Permeate quality obtained after treatment of high-strength OWM.

Parameters	Concentration in Permeates from Different Membrane Samples (mg L^−1^)		
	PES	PBM Casting-Coated	PBM Spray-Coated
COD	134.0 ± 10%	94.5 ± 7%	116.0 ± 10%
TOC	36.0 ± 11%	28.3 ± 8%	21.5 ± 9%
N–NH_4_^+^	Below detection limit	Below detection limit	Below detection limit
N–NO_3_^–^	141.2 ± 9%	144.0 ± 6%	140.3 ± 4%

## Data Availability

The data presented in this study are available on request from the corresponding authors.
